# Supplementary material for: Complex‐centric proteome profiling by SEC‐SWATH‐MS
Source: Mol Syst Biol. 2019 Jan 14;15(1):e8438. doi: 10.15252/msb.20188438 (PMC6346213; doi:10.15252/msb.20188438)
Supplement: Supplementary file 8 — Dataset EV7 [file MSB-15-e8438-s008.zip › feature_plots_string/Q06830.pdf]

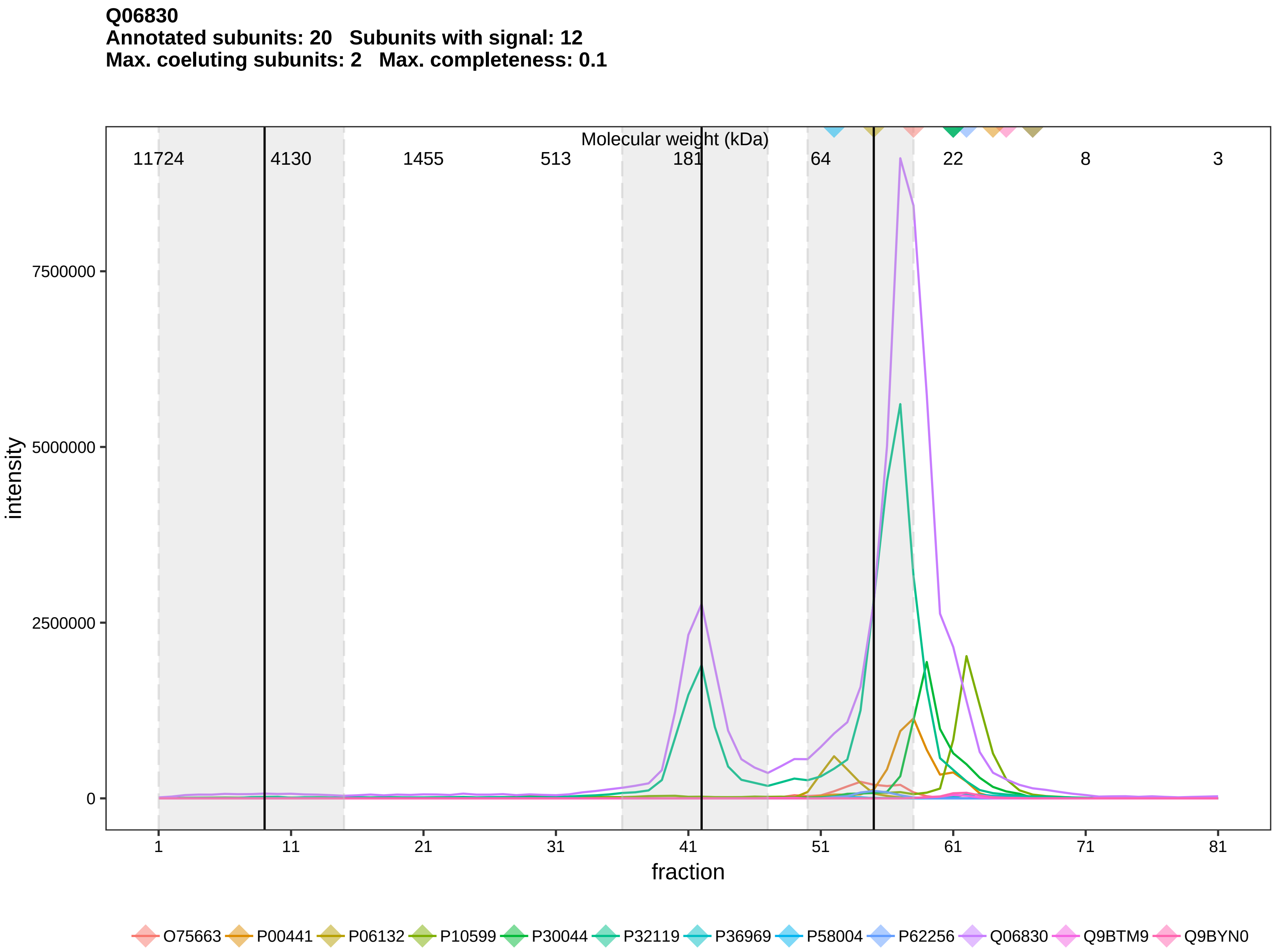
Q06830
Annotated subunits: 20 Subunits with signal: 12
Max. coeluting subunits: 2 Max. completeness: 0.1
Molecular weight (kDa)
11724
4130
1455
513
181
64
22
8
3
intensity
7500000
5000000
2500000
0
1
11
21
31
41
51
61
71
81
fraction
O75663
P00441
P06132
P10599
P30044
P32119
P36969
P58004
P62256
Q06830
Q9BTM9
Q9BYN0
